# Electromagnetically Induced Transparency-like Effect in U-Shaped Silicon Metasurfaces and Gap-Mode-Enhanced Refractive Index Sensing

**DOI:** 10.3390/s26082328

**Published:** 2026-04-09

**Authors:** Guangyue Shi, Ou Zhang, Changliang Li, Yiming Liu, Feng Luo

**Affiliations:** School of Materials Science and Engineering, Nankai University, Tianjin 300350, China; sgy@mail.nankai.edu.cn (G.S.); 1120220599@mail.nankai.edu.cn (O.Z.); 1120220583@mail.nankai.edu.cn (C.L.); 1120220591@mail.nankai.edu.cn (Y.L.)

**Keywords:** silicon metasurfaces, electromagnetically induced transparency, near-field enhancement, refractive index sensing

## Abstract

Electromagnetically induced transparency-like effects in silicon metasurfaces have attracted considerable interest due to their capability to manipulate optical resonances and improve sensing performance. In this work, a U-shaped silicon metasurface is proposed, consisting of a horizontal nanopillar supporting bright mode and two vertical nanopillars supporting dark mode. The coupling and coherent interference between the bright and dark modes lead to a pronounced EIT-like effect at specific wavelengths. By introducing nanoscale gaps between the horizontal and vertical silicon pillars, a U-shaped silicon metasurface with gap mode (UG metasurface) is formed, which induces strong near-field enhancement and is associated with reduced radiative losses, thereby improving the quality factor of the EIT-like resonance of UG metasurfaces. Two silicon metasurface samples are fabricated, and their transmission spectra are experimentally measured, showing good agreement with numerical simulations. In addition, the refractive index sensing performance of silicon metasurfaces is numerically investigated. The results show that the UG metasurface design significantly enhances the sensing capability, increasing the figure of merit from 6 RIU^−1^ to 60 RIU^−1^. The proposed silicon metasurfaces and near-field enhancement with the gap-mode mechanism provide a promising strategy for realizing high-performance optical sensing and offer valuable insights into the manipulation of electromagnetic responses.

## 1. Introduction

The electromagnetically induced transparency-like (EIT-like) effect, characterized as a narrow transparency window arising from mode coupling and coherent interference, originates from the phenomenon of electromagnetically induced transparency in quantum systems [1,2,3]. A typical signature of the EIT-like effect is the appearance of a narrow transmission peak located between two adjacent resonance dips in the transmission spectrum. Investigating this phenomenon helps to deepen the understanding of the physical mechanisms governing light–matter interactions in dielectric structures, particularly the coupling and interference processes between different resonant modes [4,5]. Moreover, owing to its narrow transparency window and strong dispersion characteristics, the EIT-like effect has attracted considerable interest for potential applications in slow-light devices, enhanced nonlinear interactions, and optical sensing [6,7].

Realizing electromagnetically induced transparency in atomic systems typically requires extremely stringent experimental conditions, such as ultralow temperatures and high-intensity laser fields [8,9,10]. In contrast, metasurfaces provide an ideal platform for investigating EIT-like effects while overcoming these demanding constraints [11,12,13,14]. Metasurfaces are artificial structures composed of periodically arranged subwavelength micro/nanostructures, in which the propagation characteristics of electromagnetic waves can be effectively tailored by adjusting the geometric parameters of the unit cell [15,16,17]. Through rational design of the unit structures and their coupling relationships, multiple resonant modes can be excited in metasurfaces. The EIT-like phenomena can be induced by engineering coupling and coherent interference between different modes [18,19,20]. Initial investigations of EIT-like phenomena in metasurfaces were primarily conducted in metallic plasmonic systems, where the effect originates from the coherent interaction between bright and dark resonant modes [21,22,23]. However, in the near-infrared region, the intrinsic Ohmic losses of metals become significantly enhanced, which leads to a reduction in the quality factor of the transparency window and weakened dispersion, thereby limiting the performance of EIT-like effects in applications such as slow light and sensing [24,25]. An effective approach to addressing this issue is to adopt all-dielectric metasurfaces, particularly by employing high refractive index materials such as silicon as the building blocks. Silicon-based metasurfaces can significantly reduce optical losses and achieve a higher quality factor (Q factor) [26,27,28]. In recent years, various configurations of silicon metasurfaces have been developed to produce EIT-like effects [29,30,31], including E-shaped metasurfaces, cross-shaped metasurfaces and T-shaped metasurfaces. These silicon-based metasurfaces have demonstrated great potential in applications such as optical sensing, low-loss optical modulators, and slow-light devices [32,33,34,35,36,37].

In this work, we design a U-shaped silicon metasurface composed of one horizontal silicon nanopillar and two vertical silicon nanopillars and theoretically investigate its EIT-like effect and underlying physical mechanism using the finite element method [38]. Unlike previous studies that reported either U-shaped silicon metasurfaces with EIT-like responses or silicon metasurfaces with gap-mode field enhancement separately, the present work establishes a directly comparable U and gap-mode design platform, in which nanoscale gaps are intentionally introduced into a U-shaped silicon metasurface to tailor the EIT-like resonance. Specifically, nanoscale gaps are introduced between the horizontal silicon pillar and the two vertical silicon pillars to construct a U-shaped silicon metasurface with gap-mode (denoted as UG silicon metasurface). The introduction of gap mode enhances the localized electric field and is consistent with reduced radiative leakage, thereby improving the Q factor of the EIT-like resonance. Two types of silicon metasurface samples are fabricated, and their transmission spectra are measured using a custom-built micro-spectroscopy optical setup, experimentally validating the structural design, numerical simulations, and the direct comparison between the U and UG metasurfaces. Finally, the refractive index sensing performance of the two metasurfaces is investigated through numerical simulations. The results indicate that the gap mode significantly enhances the refractive index sensing capability, increasing the figure of merit (FOM) from 6 RIU^−1^ to 60 RIU^−1^. The proposed silicon metasurfaces feature a simple structure and convenient fabrication process while exhibiting a pronounced EIT-like effect, demonstrating strong potential for applications in refractive index sensing. More importantly, by systematically comparing the U and UG metasurfaces in terms of resonance characteristics, field localization and sensing performance, this work provides a practical route for tuning electromagnetic responses and optimizing device performance in silicon metasurface-based EIT-like systems.

## 2. Methods

**Numerical Simulations:** Full-wave numerical simulations based on the finite element method (FEM) were performed using COMSOL Multiphysics^®^ (Version 5.6) to investigate the optical properties of the proposed metasurfaces. The dielectric properties of all materials were extracted from the built-in material library of COMSOL Multiphysics^®^. Periodic boundary conditions were implemented along the x and y axes to emulate the infinite periodic metasurfaces. A linearly polarized plane wave source was launched from above the structure and normally incident along the negative z direction, with the electric field polarized along the x direction, i.e., parallel to the horizontal silicon pillar. The wavelength was scanned from 1100 nm to 1800 nm. A nonuniform mesh refinement strategy was used, with finer meshes applied near the silicon boundaries and in the critical structural regions to accurately resolve the localized electromagnetic fields. The minimum mesh size was set to 10 nm, while coarser meshes were used in the surrounding regions to improve computational efficiency. Perfectly matched layers (PMLs) were employed at the top and bottom boundaries along the z-axis to absorb the outgoing electromagnetic waves, thereby emulating an open and infinite space and eliminating artificial reflections from the computational boundaries. This simulation configuration ensures both high computational efficiency and reliable optical analysis results.

**Sample fabrication:** Initially, a 10 nm SiO_2_ layer followed by a 300 nm amorphous silicon film was sequentially deposited on a fused silica substrate by plasma-enhanced chemical vapor deposition (PECVD) to ensure accurate deposition of the silicon layer. The deposition was carried out with a Hassrode^®^-P200A system from Jiangsu Leuven Instruments (Xuzhou, Jiangsu, China), with SiH_4_ and H_2_ serving as the precursor gases during the deposition process. Subsequently, the sample was treated with oxygen plasma to improve surface hydrophilicity. Afterward, a 240 nm-thick layer of PMMA was spin-coated onto the sample surface, followed by a prebaking process on a hot plate at 130 °C for 5 min. A conductive polymer layer was then spin-coated on top and baked at 90 °C for 2 min. The resist layer was patterned by electron-beam lithography (EBL) to define the designed structures at an acceleration voltage of 30 keV and a beam current of 43 pA, followed by a development process. The exposure process was carried out on a Helios^®^ 5CX dual-beam microscope from ThermoFisher Scientific Instruments (Waltham, MA, USA) and integrated with a pattern generator from Raith GmbH (Dortmund, Germany). A chromium hard mask for subsequent etching was then fabricated on the silicon film by thermal evaporation and a metal lift-off process. Inductively coupled plasma (ICP) etching was then performed to transfer the mask pattern into the silicon layer using a PlasmaPro 100 system (Oxford Instruments, High Wycombe, UK), with C_4_F_8_ and SF_6_ as the etching gases. After the etching process, the residual chromium mask was removed using a chromium etchant. The sample was then immersed in acetone and ultrasonically cleaned for 15 min to remove residual contaminants and obtain a clean surface. Finally, the fabricated structures were characterized using scanning electron microscopy (SEM), and the transmission spectra were measured using a custom-built micro-spectroscopy setup.

**Optical measurement:** A custom-built transmission micro-spectroscopy setup was employed to characterize the transmission of the metasurface samples. The supercontinuum white light generated by the laser source (Zolix, Beijing, China, TLS3-X150AU-G) was dispersed by a monochromator to obtain light beams with different wavelengths. The output beam exhibited good collimation and high intensity. The beam was then deflected by 45° using a mirror and directed into the measurement optical path, where it sequentially passed through an aperture and a neutral density filter to adjust the beam size and incident intensity according to experimental requirements. Meanwhile, broadband white light emitted from an LED source was guided through two beam splitters and an incident objective lens (Thorlabs, Newton, NJ, USA, LMM40X-UVV), producing a focused spot of approximately 20 μm on the sample surface. The position of the light spot was monitored using a CCD imaging system (Thorlabs, CS126CU), and the sample mounted on a translation stage was precisely adjusted so that the spot was accurately aligned with the target region of the silicon metasurfaces. Subsequently, a linear polarizer (Thorlabs, WP25M-UB) placed in front of the incident objective lens served as a polarizer to convert the incident light into linearly polarized light along the x direction. The transmission measurements were carried out under near-normal incidence. The beam was then focused onto the metasurface structure through the incident objective lens and transmitted through the sample. The transmitted light passing through the sample was collected by an objective lens located on the exit side (Thorlabs, LMM40X-UVV) and subsequently detected by an InGaAs photodetector (Zolix, DInGaAs2600-TE). Using this optical configuration, the transmission spectra of the fabricated silicon metasurface samples were recorded. The measured spectra were normalized with respect to air to remove the background response of the measurement system.

## 3. Results and Discussion

### 3.1. U-Shaped Silicon Metasurface: Structure Design and Characterization

**Figure 1:**
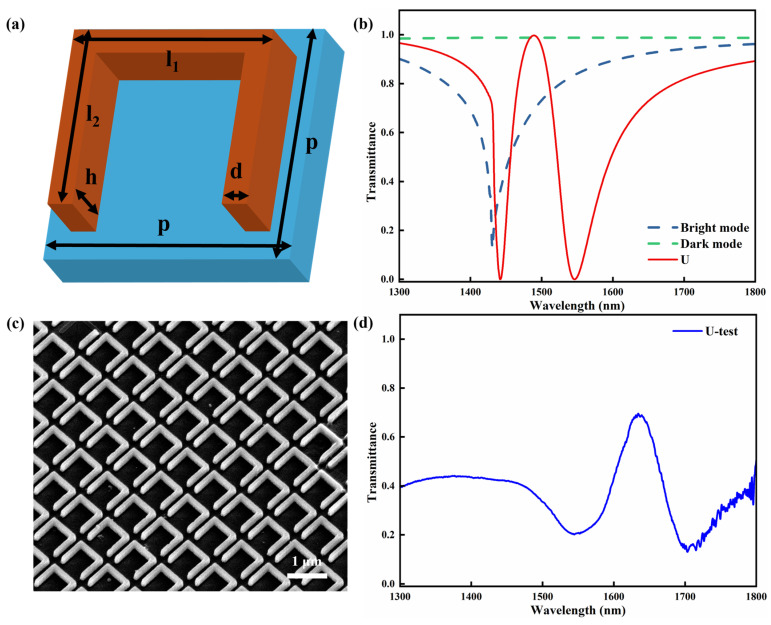
(**a**) Schematic illustration of the unit cell of the U-shaped silicon metasurface. The geometric parameters are as follows: the length of the horizontal nanopillar l_1_ = 800 nm, the length of the two vertical nanopillars l_2_ = 750 nm, the structural height h = 300 nm and the line width d = 100 nm. (**b**) Simulated transmission spectra of the U-shaped metasurface, the bright mode and the dark mode. (**c**) SEM image of the fabricated U-shaped silicon metasurface sample. (**d**) Measured transmission spectrum of the U-shaped metasurface sample.

The schematic of the unit cell for the U-shaped silicon metasurface (denoted as U) is presented in Figure 1a. The structure is composed of periodically arranged silicon nanopillars deposited on the fused silica substrate. The unit cell consists of three silicon nanopillars, including one horizontal pillar and two vertical pillars. The metasurface has a lattice period of *p* = 990 nm in both the x and y directions. Under illumination, the horizontal nanopillar couples strongly to the incident electromagnetic wave and therefore acts as a bright resonator. By contrast, the two vertical nanopillars support a weakly radiative dark mode, which cannot be efficiently excited directly by the incident wave. The excitation of the dark mode relies on near-field coupling with the bright mode. This coupling induces coherent interference between the bright and dark modes at particular wavelengths, which gives rise to an EIT-like phenomenon. Figure 1b presents the simulated transmission spectra of different structures. The red solid curve corresponds to the U-shaped silicon metasurface, whereas the blue and green dashed curves represent the horizontal silicon nanopillar (bright mode) and the two vertical silicon nanopillars (dark mode), respectively. The transmission spectrum of the horizontal nanopillar exhibits a pronounced resonance dip at approximately 1431 nm, confirming its bright-mode character. In contrast, the transmission spectrum of the two vertical nanopillars remains nearly flat and close to 1, indicating that the corresponding dark-like mode is weakly radiative. The spectrum of the U-shaped silicon metasurface exhibits a pronounced EIT-like transmission peak. The transmission dips occur near 1440 nm and 1545 nm, while the transmission peak appears at approximately 1488 nm with a transmission value reaching 0.99. According to the definition of the quality factor: Q = λ_0_ × FWHM^−1^, where λ_0_ corresponds to the wavelength at the transmission maximum, FWHM represents the spectral linewidth defined by the full width at half maximum, the FWHM extracted from Figure 1b is 65 nm, and the Q factor is 23.

We fabricated the U-shaped silicon metasurface on the fused silica substrate coated with silicon film, and Figure 1c presents the SEM image of the fabricated sample. The silicon nanopillars exhibit steep sidewalls, and the unit structures show few defects. In addition, the substrate surface appears clean and free of noticeable impurities. The custom-built micro-spectroscope setup was employed to characterize the transmission of the fabricated sample, and the measured spectrum is presented in Figure 1d. The experimental results also exhibit a typical EIT-like transmission peak located near 1635 nm, while the transmission dips appear around 1555 nm and 1700 nm. The measured results show the same trend and physical characteristics as the simulated spectrum, which verifies the reliability of the numerical simulations and theoretical analysis. It should be noted that the experimental spectrum exhibits an obvious redshift and a lower transmission than the simulated result. To further clarify the origin of this discrepancy, additional parametric simulations were performed by varying the lattice period and the vertical nanopillar lengths of the U-shaped metasurface. The influence of these geometric parameters on the resonance wavelength is discussed below. In addition, the lower transmission observed in the experiment may result from structural imperfections of the fabricated metasurface samples, deviations in the material optical constants and extra losses in the optical measurement system.

**Figure 2:**
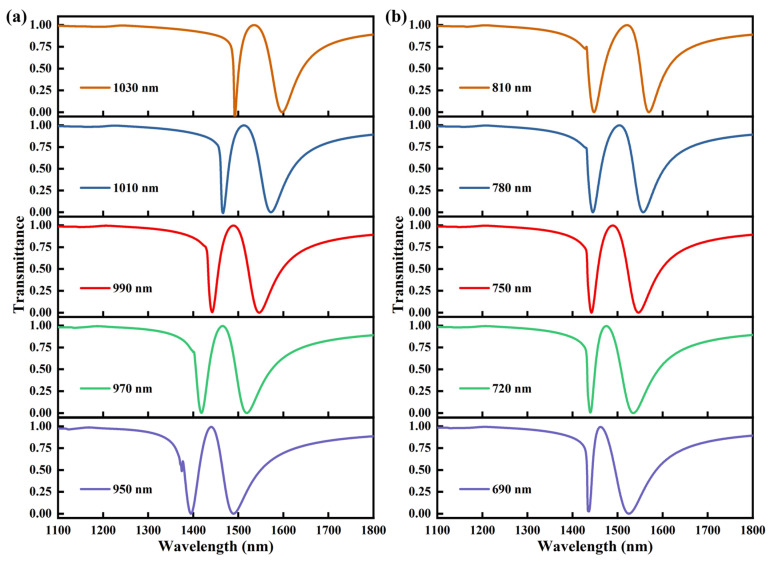
Simulated transmission spectra of the U-shaped silicon metasurface for different geometric parameters, (**a**) different lattice periods, (**b**) different lengths of the vertical silicon nanopillars.

To clarify the influence of the unit-cell geometric parameters on the EIT-like response of the metasurface, additional parametric simulations were carried out. First, the lattice period varied from 950 nm to 1030 nm with an increment of 20 nm, and the corresponding transmission spectra are presented in Figure 2a. It can be seen that the EIT-like feature is preserved throughout the investigated range, while the transparency peak shifts progressively toward longer wavelengths as the period increases. Figure 2b shows the transmission spectra of the U-shaped metasurface for different lengths of the vertical silicon nanopillars. The length varied from 690 nm to 810 nm with an increment of 30 nm. A clear redshift of the EIT-like transparency peak is observed with increasing pillar length. Physically, increasing the vertical pillar length enlarges the effective resonant path of the mode, whereas increasing the lattice period modifies the inter-unit coupling and the effective optical environment of the metasurface, both of which shift the resonance toward longer wavelengths. These results indicate that the EIT-like resonance peak is sensitive to the geometric parameters of the unit cell. Therefore, fabrication-induced deviations in the lattice period and pillar length may be one possible origin of the redshift observed in the experimental spectra, although other factors, such as differences in material optical constants and nonideal experimental conditions, may also contribute.

**Figure 3:**
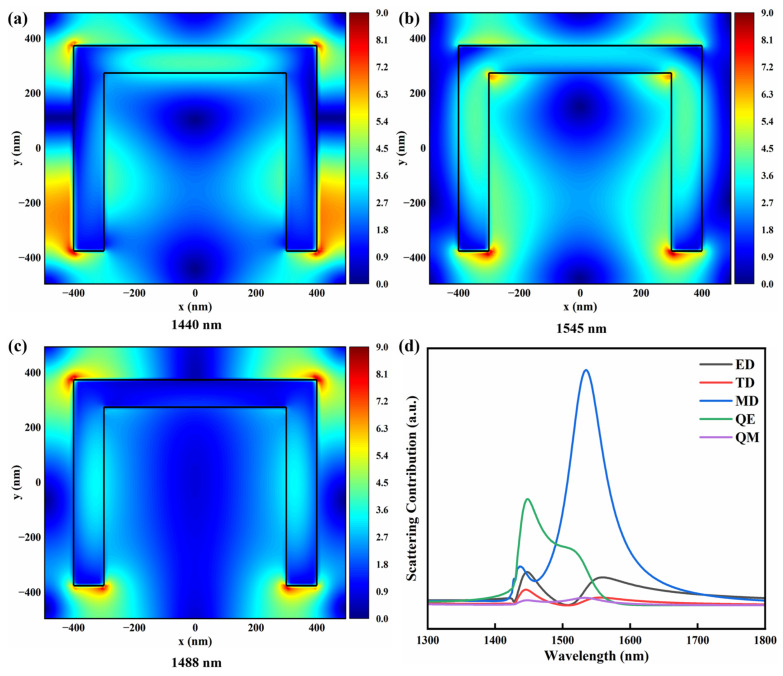
Electric field amplitude distributions in the x–y plane of the U-shaped silicon metasurface at different wavelengths: (**a**) 1440 nm, (**b**) 1545 nm, and (**c**) 1488 nm. (**d**) Multipole decomposition of the scattering power of the metasurface, showing the contributions of the electric dipole (ED), toroidal dipole (TD), magnetic dipole (MD), electric quadrupole (QE), and magnetic quadrupole (QM).

To further clarify the physical origin of the EIT-like effect, the multipole contributions and the spatial distribution of the electric field amplitude in the x–y plane were analyzed, as shown in Figure 3. The corresponding electric field amplitude pattern at 1440 nm is plotted in Figure 3a. Strong electric field enhancement can be observed at the two ends of the horizontal silicon nanopillar and at the bottom of the two vertical nanopillars, exhibiting a field distribution consistent with a characteristic quadrupole-like pattern. The corresponding multipole decomposition in Figure 3d shows a pronounced enhancement of the electric quadrupole (QE) component at this wavelength, indicating that the transmission dip mainly originates from a hybrid resonance dominated by the QE mode. As a result, the interaction between the incident light and the structure becomes stronger, increasing scattering and thereby reducing the transmitted intensity [24].

The electric field amplitude distribution at 1545 nm is shown in Figure 3b. Strong electric-field enhancement appears at the bottom of the two vertical silicon nanopillars, while a clear circulating field pattern associated with displacement-current loops is formed within the U-shaped structure, which is a typical signature of a magnetic dipole (MD) resonance. The multipole decomposition in Figure 3d further confirms that the MD contribution dominates at this wavelength. This mode exhibits strong scattering characteristics, causing a larger portion of the incident energy to couple into the resonant scattering channel, thereby suppressing the transmission.

Conversely, the EIT-like transmission peak at 1488 nm is attributed to the coupling and coherent interference between the previously described QE and MD modes. Strong electric field enhancement appears at the four corners of the U-shaped structure, as displayed in Figure 3c, together with enhanced fields inside the silicon pillars, indicating that both modes simultaneously participate in the electromagnetic response. Combined with the multipole decomposition at this wavelength, the QE and MD resonant channels are coupled, and their radiative fields interfere coherently in the far field, leading to enhanced transmission and ultimately forming the characteristic EIT-like transparency window. This observation indicates that near-field coupling between these modes enables the bright mode to excite the weakly radiative dark mode, producing coherent interference and giving rise to the EIT-like effect [18].

### 3.2. UG Silicon Metasurface: Gap-Mode Enhancement and Q Factor Improvement

The quality factor (Q factor) is an important parameter in the study of EIT-like effects, as a higher Q value generally corresponds to a narrower resonance linewidth and a spectrally selective transparency window, indicating reduced energy dissipation in the system. For EIT-like resonances, a high Q factor is particularly beneficial for applications such as refractive-index sensing, since it can significantly improve the figure of merit (FOM). To achieve a higher Q factor for the EIT-like resonance in the U-shaped silicon metasurface, nanoscale gaps were introduced between the horizontal nanopillar and the two vertical nanopillars, thereby providing an additional degree of freedom for tailoring the local field distribution and mode interaction [39,40].

**Figure 4:**
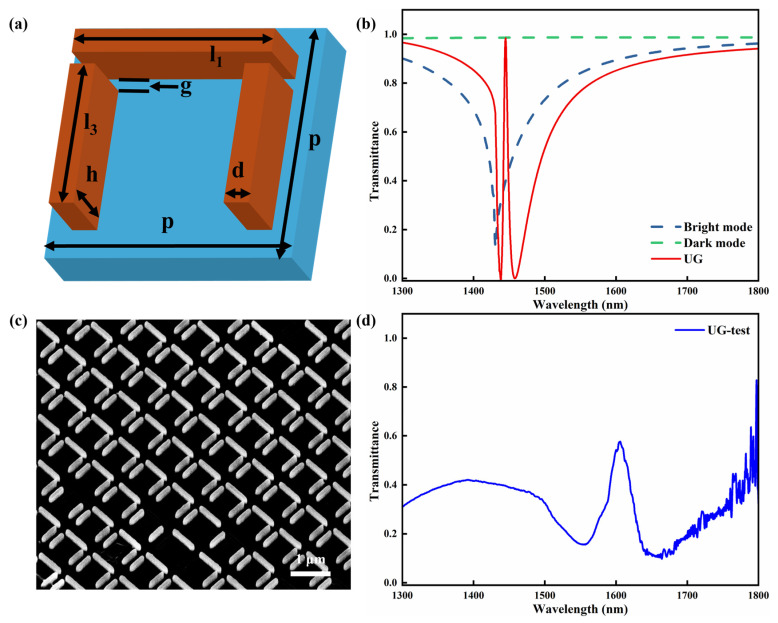
(**a**) Schematic illustration of the unit cell of the UG silicon metasurface. The geometric parameters are as follows: the length of the horizontal nanopillar l_1_ = 800 nm, the length of the two vertical nanopillars l_3_ = 600 nm, the gap between horizontal and vertical nanopillars g = 50 nm, the structural height h = 300 nm and the line width d = 100 nm. (**b**) Simulated transmission spectra of the UG metasurface, the bright mode and the dark mode. (**c**) SEM image of the fabricated UG silicon metasurface sample. (**d**) Measured transmission spectrum of the UG metasurface sample.

Figure 4a illustrates the schematic structure of the U-shaped silicon metasurfaces with gap mode (denoted as UG). A nanoscale gap of g = 50 nm was introduced between the horizontal nanopillar and the two vertical nanopillars by shortening the lengths of the vertical silicon nanopillars, while keeping the other geometric parameters unchanged. In this configuration, the horizontal nanopillar continues to support a bright mode, whereas the two vertical nanopillars support a weakly radiative dark mode. At certain wavelengths, coherent coupling and interference between the bright and dark modes result in an EIT-like effect. Figure 4b presents the calculated transmission spectra of the isolated horizontal nanopillar and the two vertical nanopillars, together with that of the coupled UG metasurface. The isolated horizontal nanopillar exhibits a broad resonance dip at approximately 1431 nm, confirming its bright-mode character, while the spectrum of the two vertical nanopillars remains nearly flat and close to unity, indicating strongly suppressed direct excitation of the dark-like mode. The red solid curve represents the calculated transmission response of the UG silicon metasurface, which exhibits a typical EIT-like feature. The transparency peak is located near 1445 nm, while the transmission dips appear at approximately 1437 nm and 1458 nm. The EIT-like transparency peak exhibits a full width at half maximum (FWHM) of approximately 6 nm, yielding a Q factor of 241. Compared with the results in Section 3.1, the introduction of the gap mode leads to a noticeable enhancement of the Q factor and a narrowing of the FWHM of the EIT-like resonance.

Figure 4c presents the SEM image of the UG silicon metasurface fabricated using the same fabrication process as that used for the U-shaped structure. The nanoscale gaps can be clearly identified, confirming that the intended gap-mode geometry was successfully realized. Nevertheless, several unit cells show incomplete or distorted features, which are likely associated with imperfections during electron beam exposure, pattern development or chromium hard mask formation. These fabrication-induced defects may affect the resonance characteristics and contribute to deviations between the measured and simulated spectra.

The transmission spectrum of the fabricated UG metasurface sample measured using the custom-built optical setup is shown in Figure 4d. A typical EIT-like response is observed, characterized by two transmission dips located near 1555 nm and 1645 nm, with a transparency peak appearing between them at approximately 1605 nm. Compared with the experimental spectrum of the U-shaped metasurface shown in Figure 1d, the FWHM of the EIT-like transparency window in Figure 4d becomes significantly narrower, which is consistent with the higher-Q behavior predicted by the simulations. Although the experimental spectrum exhibits a noticeable redshift relative to the simulated result, it preserves the same characteristic EIT-like line shape, thereby supporting the validity of the gap-engineered design. To further assess the role of geometric parameters, additional parametric simulations of the UG metasurface were carried out by varying the gap width and the lattice period. The corresponding results are used to evaluate spectral dependence on these parameters, to justify the choice of g = 50 nm, and to discuss whether fabrication-induced geometric deviations may be one of the factors contributing to the experimental redshift. In addition, both the measured Q factor and transmission amplitude are lower than those predicted by simulations. This discrepancy may arise from structural imperfections in the fabricated sample, intrinsic material loss and additional losses introduced by optical components in the measurement setup.

**Figure 5:**
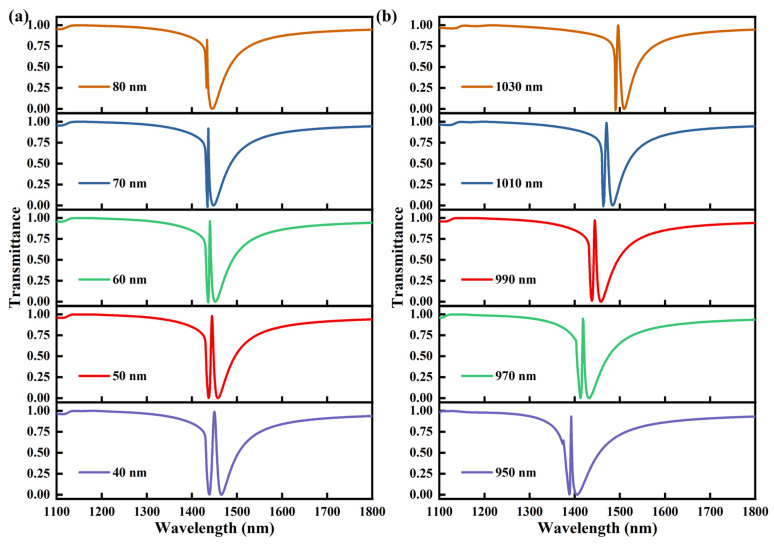
Simulated transmission spectra of the UG silicon metasurface for different geometric parameters, (**a**) different gap width, (**b**) different lattice periods.

Figure 5a,b show the simulated parametric dependence of the EIT-like response on the gap width and lattice period, respectively. As shown in Figure 5a, when the gap width increases from 40 nm to 70 nm, the FWHM of the transparency peak gradually decreases, indicating a continuous increase in the Q factor. When the gap width further increases to 80 nm, the EIT-like feature becomes degraded, suggesting that an excessively large gap weakens the coupling between the bright and dark modes. It should be noted that the Q factor does not increase monotonically with decreasing gap width. Although a smaller gap can enhance the capacitive near-field coupling and field confinement, it can also modify the eigenfrequency of the dark mode and the coupling strength between the two modes. Therefore, the highest Q factor is achieved only when the modal detuning and coupling strength are properly balanced, rather than at the minimum gap width. In this work, a gap width of 50 nm was selected as the optimized parameter by considering the resonance characteristics, fabrication feasibility and optical measurability. As shown in Figure 5b, the lattice period mainly affects the spectral position of the EIT-like resonance. When the period increases from 950 nm to 1030 nm, the EIT-like response exhibits an obvious redshift, while the overall spectral shape remains nearly unchanged. This result indicates that increasing the period may change the electromagnetic coupling between adjacent unit cells and the effective optical path of the system, thereby shifting the resonance to longer wavelengths. Therefore, one possible reason for the redshift observed in the experimental spectra, compared with the simulated results, is that the actual period of the fabricated sample is slightly larger than the designed value. Such a deviation may be introduced during electron-beam lithography, development, or etching and can lead to an overall redshift of the measured spectra.

**Figure 6:**
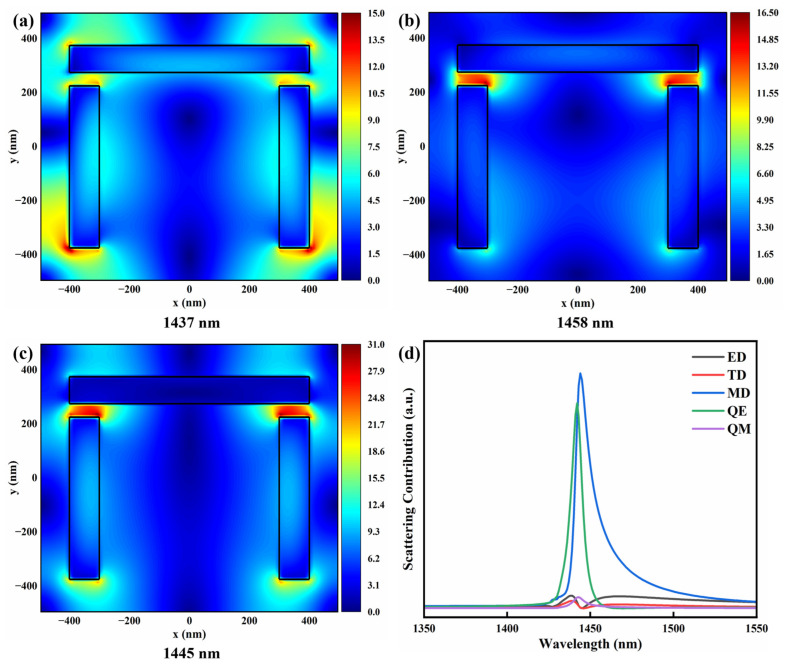
Electric field amplitude distributions in the x–y plane of the UG silicon metasurface at different wavelengths: (**a**) 1437 nm, (**b**) 1458 nm, and (**c**) 1445 nm. (**d**) Multipole decomposition of the scattering power of the metasurface, showing the contributions of the electric dipole (ED), toroidal dipole (TD), magnetic dipole (MD), electric quadrupole (QE), and magnetic quadrupole (QM).

To further analyze and elucidate the origin of the Q-factor improvement induced by the introduction of the gap mode, the electric field amplitude distributions in the x–y plane and the corresponding multipole contributions of the UG metasurface were analyzed, as shown in Figure 6. Figure 6a–c present the distributions at the two transmission dips and the EIT-like transparency peak, respectively, while Figure 6d shows the corresponding multipole decomposition. The electric field inside the gap is significantly enhanced in all three cases, and the field intensity within the gap is noticeably higher than that in other parts of the unit structure. These results demonstrate that the gap mode induces significant near-field enhancement, with electromagnetic energy effectively confined within the gap, thereby suppressing radiative losses. Moreover, the introduction of the gap enables energy exchange inside the structure to be dominated by displacement currents and capacitive near-field interactions. In addition, compared with the U-shaped metasurface, the multipolar response of the UG structure becomes much more spectrally concentrated, with the resonance dominated by narrow MD and QE features, while the contributions from other channels remain relatively weak. This behavior is consistent with reduced radiative leakage and a stronger confinement of electromagnetic energy within the structure, which together explain the significantly enhanced Q factor of the EIT-like resonance in the UG metasurface. Therefore, the introduction of the gap mode not only strengthens the near-field confinement but also provides an effective way to tailor the scattering channels and improve the resonance quality of silicon metasurfaces-based EIT-like systems [41,42,43].

### 3.3. Coupled-Oscillator Model Analysis

**Figure 7:**
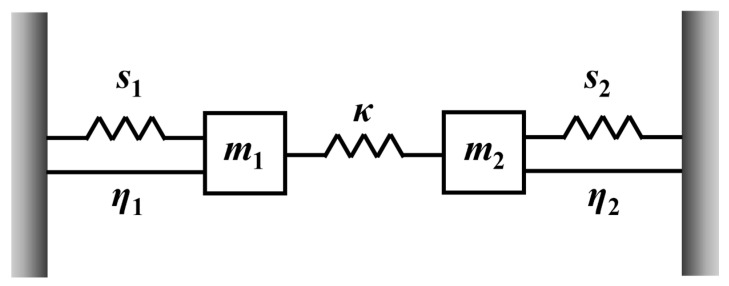
Schematic of the coupled-oscillator model.

The EIT-like phenomenon can be interpreted using a coupled-oscillator model, as schematically illustrated in Figure 7. In this framework, two oscillators with effective masses *m*_1_ and *m*_2_ are introduced to represent the resonant elements of the EIT-coupled system. The parameters (*η*_1_, *η*_2_) and (*s*_1_, *s*_2_) denote the damping rates and resonance strengths of the two oscillators, respectively. When only one oscillator is driven by an external harmonic force, it indicates that only one resonant element can be efficiently excited by the incident field and therefore behaves as the bright mode, whereas the other element is only weakly excited and can be regarded as the dark mode. This scenario corresponds to an EIT-like system governed by bright–dark mode coupling [44,45].

In the presence of an incident electromagnetic field with electric-field amplitude $E=E_{0}e^{i\omega t}$, the displacements of the two resonators are denoted by *x*_1_ (*t*) and *x*_2_ (*t*), respectively. The dynamical responses of the two resonators then satisfy the following set of coupled differential equations [46,47]:

(1) x¨1t+η1x˙1t+ω12x1t+κ2x2t=s1Em1

(2) x¨2t+η2x˙2t+ω22x2t+κ2x1t=s2Em2

Here, (*ω*_1_, *ω*_2_) represent the intrinsic resonance frequencies of the two oscillators, respectively, while κ denotes the coupling strength between them. The interaction strengths between the two oscillators and the incident electromagnetic wave are characterized by (*g*_1_, *g*_2_). Furthermore, the transmission can be derived as:

(3) $$t(\omega )=\frac{4\tilde{n}(\omega )e^{i\phi (\omega )}}{{(1+\tilde{n}(\omega ))}^{2}-{(1-\tilde{n}(\omega ))}^{2}e^{2i\phi (\omega )}}$$

(4) $$T(\omega )=|t(\omega ){|}^{2}$$

**Figure 8:**
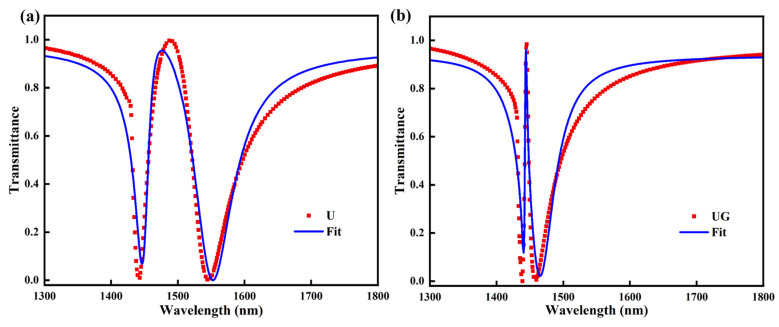
Coupled-oscillator fitting of the EIT-like transmission spectra for the (**a**) U metasurface and (**b**) UG metasurface. The red squares represent the simulated transmission spectra, and the blue solid curves denote the fitted results obtained from the coupled harmonic oscillator model.

According to the above formulation, the transmission spectra of the U and UG metasurfaces can be fitted, with the corresponding fitting results shown in Figure 8. In addition, the fitted parameters are summarized in Table 1.

Figure 8 shows that the coupled-oscillator model reproduces the EIT-like transmission responses of both the U and UG metasurfaces well. In both cases, the fitted curves agree closely with the simulated spectra, including the two resonance dips and the transparency peak between them, confirming that the EIT-like behavior can be understood as the interference between bright mode and dark mode. Compared with the U metasurface, the UG metasurface exhibits a much narrower and sharper transparency window, indicating a stronger and more ideal EIT-like interference effect. This trend is further supported by the fitted parameters listed in Table 1. For the UG metasurface, the damping rates decrease from *η*_1_ = 6.134 THz and *η*_2_ = 0.073 THz in the U structure to *η*_1_ = 3.870 THz and *η*_2_ = 0.062 THz, indicating reduced effective energy dissipation, especially for the bright mode. Meanwhile, the modal detuning decreases significantly from |∆*ω*| = 5.904 THz to 2.628 THz, meaning that the two effective modes become spectrally closer and can couple more efficiently to produce a sharper destructive-interference window. In addition, the excitation ratio of the second oscillator decreases from *r* = *g*_2_/*g*_1_ to nearly zero, suggesting that the second oscillator in the UG metasurface behaves more like an ideal dark mode with much weaker direct coupling to the incident field. Therefore, the Q-factor enhancement of the UG metasurface originates from the combined effects of lower damping, smaller modal detuning, and a weaker radiative dark-like mode, which together result in a much narrower EIT-like resonance linewidth and hence a significantly higher Q value.

### 3.4. Refractive Index Sensing

**Figure 9:**
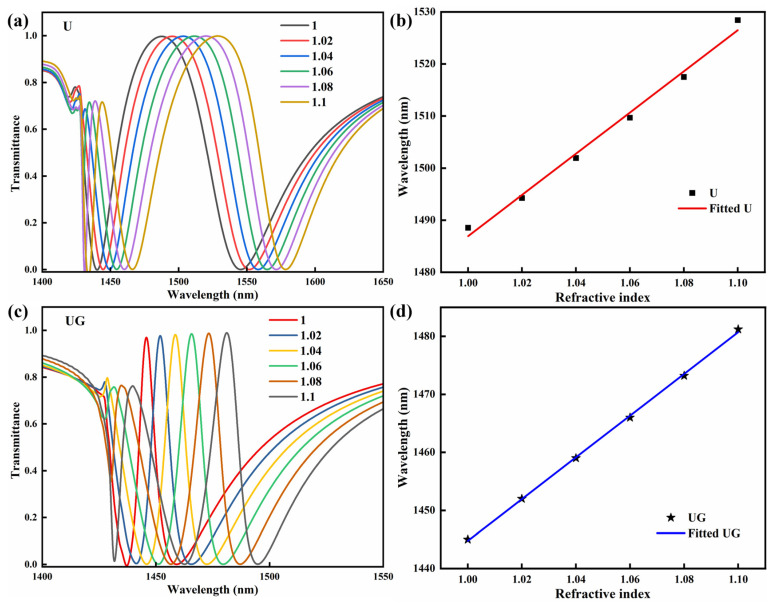
(**a**,**c**) Simulated transmission spectra of U-shaped and UG metasurfaces with refractive indices ranging from 1.0 to 1.1. (**b**,**d**) EIT-like peak wavelength as a function of refractive index for the corresponding metasurfaces. Squares denote the simulated data, and solid lines represent the linear fits.

Because of the narrow transparency window, EIT-like responses are highly sensitive to variations in the surrounding refractive index, making them promising for refractive index sensing. To investigate this sensing capability, numerical simulations were carried out to evaluate the transmission behavior of the two silicon metasurfaces under different refractive index conditions. In the simulations, the refractive index of the surrounding environment varied from 1.0 to 1.1, and the resulting transmission spectra are presented in Figure 9. The results for the U-shaped silicon metasurface and the UG silicon metasurface are shown in Figure 9a,c, respectively. As the refractive index increases, the transmission of the EIT-like transparency window exhibits a slight increase. Meanwhile, even a small change in the refractive index leads to a noticeable shift in the EIT-like resonance toward longer wavelengths.

Two important parameters used to characterize optical resonance sensing are the FWHM of the resonance and the shift in the resonance wavelength caused by a unit variation in the refractive index, which is defined as the refractive index sensitivity (S) [48]. Based on these two quantities, another key parameter for evaluating sensing performance, the figure of merit (FOM), can be calculated according to FOM = S × FWHM^−1^ [49]. The dependence of the EIT-like resonance wavelength on the refractive index of the surrounding medium for the two metasurfaces is presented in Figure 9b,d. It can be observed that the resonance wavelength exhibits a linear dependence on the refractive index. The black squares represent the simulated data, while the solid lines correspond to the linear fitting results. The slope obtained from the linear fit gives the refractive index sensitivity.

According to the calculations, the designed U-shaped silicon metasurface exhibits a sensitivity of 395 nm/RIU and a FOM of approximately 6 RIU^−1^. In comparison, the UG silicon metasurface shows a sensitivity of 360 nm/RIU, while its FOM reaches approximately 60 RIU^−1^. Although the sensitivity of the UG metasurface is slightly lower than that of the U-shaped metasurface, its FOM is improved by one order of magnitude. This apparent trade-off can be understood from the nature of the gap-mode-enhanced EIT-like resonance. In the UG structure, the electromagnetic field becomes more strongly confined within the nanoscale gap and the adjacent silicon region, which enhances the local mode confinement and reduces radiative leakage, but at the same time slightly decreases the effective interaction of the resonance with the bulk surrounding medium. As a result, the resonance wavelength shift induced by a unit refractive-index variation becomes slightly smaller. However, the FWHM of the EIT-like transparency window is dramatically reduced due to the significantly enhanced Q factor, and this narrowing effect dominates the FOM. Therefore, the much higher FOM of the UG metasurface mainly originates from its ultranarrow resonance linewidth rather than from an increase in sensitivity.

As summarized in Table 2, the present UG metasurface shows competitive sensing performance among simple dielectric EIT-like metasurfaces. Although its absolute sensing metrics are still lower than those of some highly optimized dielectric metasurfaces, the introduction of a nanoscale gap in a simple U-shaped silicon platform leads to a clear improvement in FOM from the U design to the UG design. Together with the field analysis, multipole decomposition, and coupled-oscillator fitting, these results show that gap engineering is a feasible strategy for improving the sensing performance of silicon-based EIT-like metasurfaces.

## 4. Conclusions

In summary, we investigated the electromagnetically induced transparency-like effect in silicon-based metasurfaces using a directly comparable U-to-UG design platform. The EIT-like effects emerge at particular wavelengths due to the coupling and coherent interference of the bright and dark modes. By introducing nanoscale gaps into the U-shaped silicon metasurface, the local electromagnetic field becomes more strongly confined, resulting in a narrower resonance linewidth and a significantly enhanced Q factor. The proposed structures were further supported by experimental characterization. Numerical simulations were performed to evaluate the potential of the EIT-like effect for refractive index sensing. The results demonstrate that the incorporation of the gap mode not only enhances the Q factor but also markedly improves the refractive index sensing capability of the metasurfaces, with the FOM improving from 6 RIU^−1^ to 60 RIU^−1^. More importantly, this work demonstrates that gap engineering provides an effective and physically transparent strategy for regulating EIT-like resonances and improving sensing performance in simple silicon metasurface platforms. Beyond refractive-index sensing, the proposed design concept may also be extended to integrated photonic devices requiring compact high-Q dielectric resonators, such as on-chip spectral filtering, low-loss optical modulation and enhanced light–matter interaction. These results offer useful guidance for the design of compact and tunable silicon-based metasurfaces for integrated photonics applications.

## Figures and Tables

**Table 1:** Fitted parameters of the coupled-oscillator model for the U and UG metasurfaces.

	U	UG
*ω*_1_	197.216 THz	205.021 THz
*ω*_2_	203.120 THz	207.649 THz
|∆*ω*| = |*ω*_2_ − *ω*_1_|	5.904 THz	2.628 THz
*η*_1_	6.134 THz	3.870 THz
*η*_2_	0.073 THz	0.062 THz
*r* = *g*_2_/*g*_1_	0.1135	≈0

**Table 2:** Comparison of sensitivity, Q factor and FOM for the metasurfaces in this work and several reported dielectric metasurface refractive-index sensors.

Structure	Sensitivity (nm/RIU)	Q Factor	FOM	Reference
Inversely tilted elliptic silicon nanopillars	122.2	415	-	[50]
H-shaped silicon disks with tilted splitting	262	>10^4^	2183	[36]
U-shaped silicon metasurface	203	130	29	[29]
Silicon asymmetric nanobar pairs	612	~200	85	[51]
U-shaped metasurface of this work	395	23	6	
UG metasurface of this work	360	241	60	

## Data Availability

The original contributions presented in this study are included in the article. Further inquiries can be directed to the corresponding author.
